# Rewarding journeys: exploring medical students’ learning experiences in international electives

**DOI:** 10.1080/10872981.2021.1913784

**Published:** 2021-04-08

**Authors:** Rintaro Imafuku, Takuya Saiki, Kaho Hayakawa, Kazumi Sakashita, Yasuyuki Suzuki

**Affiliations:** a Medical Education Development Centre, Gifu University, Gifu City, Japan; b Department of General Paediatrics, National Centre for Child Health and Development, Tokyo, Japan

**Keywords:** International electives, international education, academic contact situation, cultural adaptation, language management theory, short–term program, social interaction

## Abstract

International electives are recognized as a high-impact practice in clinical education. However, medical students’ actual learning experiences during electives have not been explored fully. Specifically, drawing on language management theory, this exploratory case study investigates students’ perceived learning outcomes and the managing processes by which they gained benefits from cross-cultural learning experiences in international electives. Written reflective reports in a series of e-portfolios were collected from 43 Japanese medical students who participated in a four-week international elective. Moreover, to further explore their emotions and the reasons behind adopting a particular adjustment behaviour, follow-up interviews with 12 students were undertaken soon after they returned home. Using reflexive thematic analysis, the qualitative data were analysed. Their perceived learning outcomes were categorized into seven themes: medical knowledge and skills, communication, career management and development, international healthcare, society and culture, medical education, and personal development. During the programme, they experienced linguistic, sociolinguistic, and sociocultural difficulties, and attempted to overcome them by employing various adjustment strategies, such as meaning-focused coping, social relationship building, management of learning opportunities, communication management, and developing approaches to learning. Managing problems in academic contact situations is not a linear process; it is iterative and cyclical. Since the combination of several strategies was needed depending on the situation, the management process is a context-dependent and complex phenomenon. The findings in this study provide new insights into student participation in short-term international elective programmes in order to develop academic and social support strategies for educators at both home and host institutions.

## Introduction

Globalization creates key challenges in current healthcare and medical education, involving issues of cultural diversity including cultural history, language, religion, and gender. Given these dynamic and complex issues in a modern society, educational programmes must be developed that address issues pertaining to globalization and learners’ intercultural competence[1]. The most common approach to internationalization in higher education is student mobility, such as encouraging students to go abroad or receiving international students for part of their training [2–4]. Even from their international experiences in short-term courses, students perceived that their personal development, global perspectives, and subject–specific knowledge benefitted[5].

In medical education, global health electives provide significant benefits to medical students, both personally and professionally, including communication skills, awareness of social determinants of health, clinical reasoning, cultural competence, and leadership [6–10]. Valuable experiential learning in a cross-cultural context trains physicians to treat patients in an increasingly interconnected world which influences professional identity formation[11]. Many medical students eventually achieved satisfactory adjustment and meaningful learning in a new cultural context [8,12].

As a result of spending time in a new culture, international students encounter a range of life changes. Particularly, in a new and unfamiliar culture, international students are faced with many constraints, including language barriers, learning difficulties, differing expectations of educators, and dealing with sociocultural stressors [13,14]. The physical and psychological symptoms of culture shock include tiredness, insomnia, loneliness, homesickness, and lack of confidence[15]. Previous studies on medical education identified that international students’ unmet learning needs and both academic and non–academic factors negatively affected their lives in a new cultural context [13,16–18].

To overcome these challenges, several types of coping strategies are employed [14,19]. For example, problem-focused coping targets the actual interaction either by changing something about oneself or the demands of the environment. Emotion-focused coping targets the emotional distress caused by the interaction through avoidance, distraction, or reassurance-seeking. Meaning-focused coping involves active cognitive restructuring of the perceived meaning of the situation by trying to see the positive side of things, considering alternative views, or drawing on personal values to reinterpret the situation.

However, few studies have addressed actual experiences and learning processes of medical students during international electives. Using an exploratory case study methodology [20], this study attempts to provide an insightful account of Japanese medical students’ lived learning experiences during a short-term international elective programme through the analytical lens of language management during academic contact situations[21]. In language management theory, comprehensive competence that integrates linguistic, sociolinguistic, and sociocultural aspects is essential for maintaining cross–cultural interactions in academic contact situations [21–23]. Specifically, the processes of managing problems consists of five stages [24]: i) deviations from norms occur in a communicative situation, ii) such deviations are noted, iii) noted deviations are evaluated, iv) adjustment is planned, and v) the adjustment is implemented. All communication problems and phenomena occurring in contact situations can be related to the above–mentioned stages of the management process.

Based on the theoretical framework, we developed the following research questions pertaining to learning outcomes and processes in the electives: 1) What learning outcomes did Japanese medical students perceive after international electives? 2) What difficulties did they encounter? And 3) How did they cope with these difficulties during the electives?

## Methods

### Study context

In contrast to global health electives in western countries, such as the US and Canada, the electives in Japan are generally undertaken in developed countries. In other words, the major international elective destinations for Japanese medical students are European and North American countries. International electives at Gifu University School of Medicine are optional modules of four or eight weeks in the final year clinical clerkship programme. Goals and expectations of the international electives were to observe, understand and experience clinical practices in different cultural contexts, which were explained in the first session of the preparatory course and described on the syllabus. Evaluation forms that would be filled out by the host institution were also shared in order for the students to understand the assessment criteria. Through this elective programme, 10–15 medical students visit foreign countries every year to study clinical medicine and gain cross-cultural experience in a clinical setting.

Gifu University requires medical students who want to take international electives to have a minimum TOEFL (Test of English as a Foreign Language) (iBT) score of 79 and complete pre-departure preparative education. A pre-departure course consisted of five 4-hour extracurricular sessions and focused on English communication in relation to basic clinical skills. Specifically, we invited a general practitioner from the UK who was teaching medical education at a Japanese university. In the class, the students learned how to conduct history taking, physical examination and case presentation in English. They also engaged in role-play with simulated patients who were international students at Gifu University. Although the first session introduced limitations and cultural issues during the electives, it was not discussed much throughout the course. In the fifth session, English OSCE (Objective Structured Clinical Examination) was conducted to assess their clinical reasoning and health communication skills, using patient scenarios. The selection for participants in the electives was based on the TOEFL score and performance of English OSCE.

Countries visited by students are also limited to a region that the Ministry of Foreign Affairs of Japan indicates is sufficiently safe for travel. Methods for finding host institutions include established partner institutions, personal connections of professors, and online applications for programmes available to external students.

An e-portfolio was adopted for the international elective students by the home institution to not only enhance students’ reflective learning but also confirm their safety while they are overseas. As this was part of the formative assessment of their learning, they were required to submit their weekly written report on what they could and could not do, what they felt at that time, and what they learned through their experiences in hospital and daily life.

The students were given a summative assessment of their elective performance by the clinical supervisor at the host institution in the final week on medical knowledge, physical examination, data gathering and analysis, patient rapport, presentation skills, attitude as a team member, and so forth. After returning to Japan, students needed to submit a final written report which summarized what they had experienced and learned through their international electives. This final report was not included in the summative assessment for the electives, but rather was used for the formative assessment for their further clinical education. As it was to be written after sharing the summative assessment performed at the host institution, it would be an important data source to see some perception changes with external input.

### The research team

The research team consisted of four members with health-profession backgrounds (two paediatricians, one general practitioner, and one dentist) and one social scientist. We had a range of qualitative research experiences, and the analytical process was led by a member of the research team with methodological training and expertise in qualitative research (RI). All team members were coordinators of international electives and pre-departure programmes at Gifu University. RI, TS, and KS had experiences studying abroad for their degrees or clinical education in a certain period. Acknowledging members’ prior experiences, beliefs, and current educational roles enabled us to work together collaboratively and enhance the rigour of the qualitative analysis.

### Data collection

This study purposively selected 43 of 59 medical students at Gifu University School of Medicine who had participated in 4-week international electives from 2015 to 2019. We excluded students who had previous experiences of long-term overseas stays of six months or longer and/or of participation in an international educational program. In other words, students who had not previously stayed overseas for an extended period were included to delineate the processes of intercultural adjustment by ‘laypersons’.

This study collected a series of written reflective reports from 43 students, including the weekly e-portfolios they maintained during their electives and reports written after returning to Japan, in which their experiences, perceptions of learning, and feelings about the international electives were described in detail. Although the written student reflections presented deep enough data to explore the study’s research purposes, follow–up interviews with 12 students (i.e. Students 10–17, 19, and 22–24) were also conducted soon after they returned to Japan, to further explore their emotions and the reasons why they adopted a particular coping behaviour towards challenges they faced during their stay overseas. The criteria for selecting the students for interviews were based on the descriptions of distinctive cross-cultural experiences in e-portfolios, the countries visited, and their specialty rotated in addition to their availability and willingness to participate in the interview. The interview was semi-structured and conducted one-on-one in person. During the interviews, using the written reflective reports as a stimulus, we asked probing questions, including ‘*Please share what experience you think was most challenging; how did you feel at that moment?’*; ‘*How did you cope with the problem(s)?’*; ‘*What support did you get at the time?’*; ‘*“What would you need to work on in order to improve your experience of the international elective if you were to do another one in the future?’* and so forth. These interviews lasted 40 minutes.

### Data analysis

This study employed reflexive thematic analysis to analyse the qualitative data elicited from written reflective reports and transcripts of follow-up interviews [25,26]. First, to achieve familiarization with the data, the researchers systematically reviewed the data to better understand its content. Then, the data was broken down into small units according to meanings, actions, events, or ideas expressed by the participants. Each of these distinct units was coded and grouped into more abstract categories through the comparison of similarities and differences. These phases were repeated in an iterative procedure to ensure that the researchers’ interpretation was congruent with the presented data. It involved developing a detailed analysis of each theme, working out the focus of each theme, and determining the story of each theme to define informative names for them. It was also important to contextualize the analysis in relation to existing literature. The Japanese interview excerpts presented in this study were translated into English by the first author.

To enhance the reliability of the qualitative analysis, two researchers (RI and TS) were independently involved in coding and categorizing the data, before cross–checking their data interpretation and analysis. The preliminary findings of the analysis were discussed by all the members of the research team, including KH, KS, and YS, to establish the credibility and dependability of the data analysis.

### Ethical considerations

Ethical approval was obtained from the Gifu University Ethics Committees (No. 25–367). Confidentiality was assured for the contents of students’ reflective writing and subsequent interviews.

## Findings

### Student demographic

In 2015–2019, 43 students participated in this study. The profiles of the students are shown in Table 1 in relation to their English proficiency, the country visited, and their specialty rotated.

**Table 1.** Japanese medical student profiles (*n* = 43)

Sex	Male (27), Female (16)
English proficiency (TOEFL iBT)	100 < (=ITP600 <) (0) 90–99 (=ITP 577–597) (17) 79–89 (=ITP 550–573) (26)
Country	Australia (22), Canada (8), USA (8), Thailand (2), Austria (1), France (1), New Zealand (1)
Specialty rotated	Paediatrics (7) [Surgery (3)], Oncology (6) [Surgery (2)], Cardiology (4) [Surgery (1)], Family medicine (3), Dermatology (2), Gastrointestinal med.(2),Haematology (2), Infectious disease (2), General medicine (2), Liver transplant surgery (2), Nephrology (2), Radiology (2),Drug & Alcohol med. (1), Geriatrics (1), Neurology (1), Orthopaedic surgery (1), Palliative care (1), Rehabilitation med. (1), Urology [Surgery] (1)

### Overview of students’ management process in academic contact situations

**Figure 1.**
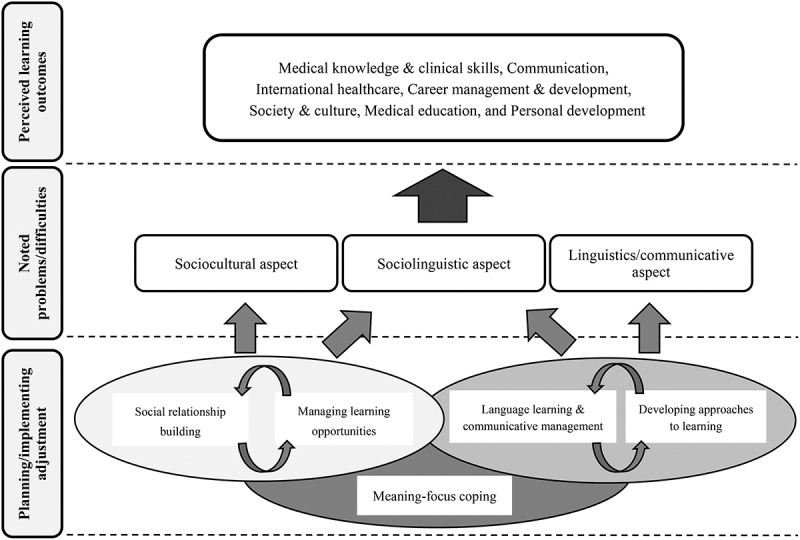
Overview of students’ management process in academic contact situation

By qualitatively analysing students’ reflective data, this study conceptualized a series of their learning experiences in international electives over four weeks, in terms of perceived learning outcomes and adjustment strategies for noted problems (Figure 1).

Figure 1 shows that learning outcomes resulting from overcoming linguistic, sociolinguistic, and sociocultural difficulties in cross-cultural experiences can be grouped into seven themes: medical knowledge and skills, communication, career management and development, international healthcare, society and culture, medical education, and personal development. Among the management strategies employed by students, meaning-focused coping is fundamental to other four strategies. Language management and developing approaches to learning were interconnected when addressing linguistic and sociolinguistic problems, whereas managing learning opportunities and social relationship building were interconnected when addressing sociolinguistic and sociocultural problems.

In the following sections, students’ experiences and perceptions at each stage are described in detail.

### Perceived learning outcomes of international electives

Table 2 shows the categories of their perceived learning outcomes with verbatim examples. Students perceived that in the electives they could not only learn communication in English or other languages but also medical knowledge, career management and development, and an international perspective on healthcare systems.

**Table 2.** Learning outcomes of international electives perceive by students

Theme	Quotations
**Medical knowledge and clinical skills**	
Medical knowledge	In Nephrology, it is important to understand the patient’s condition by fully considering the changes in every examination result. The supervisor always provided me with a detailed explanation of even the complicated cases. … I could learn a lot clinically, thanks to the clinical teachers’ detailed instructions. (Student 28)
Epidemiological differences	During the ward rounds, I could watch and learn about uncommon diseases in Japan, such as Dengue fever, Toxoplasmosis, and Bacterial dysentery. (Student 19)
Clinical skills	I had an opportunity to cannulate and draw blood, which I rarely did in Japan. … Although I couldn’t execute the procedure on patients perfectly, it’s a good experience. I realised it seemed possible theoretically but was hard to perform practically. (Student 14)
**Communication**	
English or other language	The chance to go through the history in English every day made me confident in my English language proficiency. I will be able to communicate with English-speaking patients in Japan without any problem. (Student 6)
Doctor-patient relationships	Doctors in medical oncology devoted a lot of time communicating with patients daily. I was moved by the doctors’ efforts to determine the best care for the patients through regular discussions with those suffering from serious diseases. (Student 17)
Interprofessional communication	Nurses and social workers were actively involved in taking history and patient care, which was not common in Japan. There was no hierarchical relationship among health professionals. … As issues of alcohol and drug use are critical for patients after discharge, interprofessional collaboration is importanrt. (Student 8)
**International healthcare**	
Healthcare system and policy	In Quebec, CLSCs (C*entre Local de Services Communautaires*), which are free clinics run by the government contribute to providing health and social services effectively in the society (not only remote, but also urban areas). In Japan, the family physician provides home-visit medical care services mainly in suburbs and remote areas. … I consider this system to be a good way of enhancing community medicine in Japan. (Student 39)
**Career management and development**	
Sense of professional identity	I learned the importance of enhancing the sense of professional identity. I observed that doctors remembered the clinical trial results, sample sizes, regions, publication years, and journal names precisely. Based on knowledge from previous clinical trials, they intensively discussed treatment for patients. (Student 6)
Work-life balance	In Vienna, people finished work on time and subsequently enjoyed music at a concert or a café, pictures at an art gallery, and discussed literature with their friends. Watching the citizens enjoy life, I had concerns about life in Japan where people are always hard-pressed for time and tend to overwork. (Student 16)
**Society and culture**	
Healthcare in multicultural society	Hospitals in the US offer a multicultural environment because doctors from China, Korea, India, African, European countries work together. Patients also come from various cultural backgrounds and the demand for medical interpreters is very high. Such multicultural environment allows people to share different perspectives on matters. (Student 18)
Multilingualism	Around 10% of patients spoke Chinese in Australia. Of them, many elderly people could not speak English and we needed medical staff or family members who could speak both languages. … I focused on improving my English language proficiency. However, I realised that Chinese was an equally important language in healthcare. (Student 7)
**Medical education**	
Educational system	Mentor system is well-established in the US. Every resident or fellow had a couple of mentors. A resident told me that he requested for the doctor, who was his role model, to be his mentor. (Student 11)
Educational strategies	In Thai, medical students read many research articles in international journals. In an EBM (Evidence-Based Medicine) class we discussed the best ways of treatment and prognosis of hypertensive patients based on evidence from clinical research and then logically analyzed the process of reaching our conclusions. This was an excellent opportunity to experience EBM education, which I never learned in this way before. (Student 27)
**Personal development**	
Active/lifelong learning	I realised that, under any circumstance, what you gain from experiences depends on your enthusiasm and proactive involvement in practice. This attitude is also essential in practicing as a doctor in future. (Student 15)

Knowledge and skill acquisition were achieved through observation of the supervisor’s detailed explanation of cases and engagement with tasks such as history taking, cannulation, and venepuncture. Student 19 mentioned that they had the opportunity to manage diseases that are rarely seen in their home country (i.e. Japan) due to epidemiological differences, including dengue fever, toxoplasmosis, and bacterial dysentery.

In the theme of communication, students perceived that their proficiency in English or other languages had improved through interaction with people in a clinical setting and daily life in an overseas country. Moreover, as Student 17 commented, observations of doctor-patient communication and interprofessional collaboration in patient care were an important opportunity to learn health communication.

Students observed a different healthcare system directly and reflected on their own country’s healthcare system. Comparing two healthcare systems led them to consider what the ideal system would be in their home country, such as Student 39’s reflection on the community-based medicine systems in Japan and Canada.

Career management and development was another of their perceived learning outcomes. Students realized the importance of assuming a professional attitude as a medical doctor, as Student 6 commented. Moreover, positive observations of the Viennese lifestyle led Student 16 to consider burnout and satisfaction with work-life balance among physicians in Japan.

In the theme of society and culture, students were concerned with how they could work effectively in a culturally diverse environment. For example, Student 18 discovered the advantages of multicultural work environments, and Student 7 reflected on multilingualism in healthcare through observing medical staff using Chinese as a medium of communication with patients in an English-speaking country.

International electives were an opportunity for students to consider which effective educational strategies could be adopted in the Japanese educational system after they were exposed to different approaches, such as a well-established mentorship system (Student 11), and the requirement for critical reviews of clinical research papers in evidence-based medicine education (Student 27). Students’ experiences of surviving in a different cultural environment helped them develop personally in terms of self-confidence and a proactive and self-directed attitude towards learning even though it was a short-term course (i.e. four weeks).

As Table 2 shows, students perceived that they gained some benefits from the international elective experience. To maximize learning experiences in international electives, we have to further explore the process by which students reached their positive perceptions of learning, specifically what difficulties/problems they noted and how they coped with these problems.

### Noting and evaluating problems/difficulties in contact situations

The details of these themes of noted difficulties, with illustrative quotes, are provided in Table 3. The qualitative data indicated that most problems were noted during the first half of electives, and adjustments were planned and implemented subsequently.

**Table 3.** Noted problems/difficulties in academic contact situations

Theme	Quotations
**Linguistic/communicative aspect**	
Listening	I could express what I wanted to during one-to–one interactions with clinical teachers, but, it was difficult to follow conversations between doctors in the ward and ICU rounds. In fact, I could grasp only around 30–40% of these conversations. I was shocked at how little I understood in the first few days. (Student 34)
Speaking and expression of opinion	[Expression of the detailed nuances] When the doctor questioned me about things like, ‘What do you want to do today?’ expecting my self-directed attitude, I could only respond with, ‘I want to observe surgery.’ I wanted to express more but I could not do so. I was very frustrated with my English. (Student 34)
	[Self-expression] I could not answer questions of the medical staff about clinical issues properly and coherently because I could not fully express everything I had in mind in English. This upset me and I sometimes made me doubtful about my ability to spend time productively in the US. (Student 41)
	[Presentation/explanation] I was so nervous that my voice and hands trembled when I conducted my first medical interview with a patient. My mind went blank and I was at a loss for words. When I was reporting on the patient condition to the doctor, his facial expressions made his discomfort with my English obvious. (Student 6)
Vocabulary/abbreviation	There were a high number of abbreviations compared to Japan. As there were often drugs with different brand-names on patient notes, I was confused and had difficulty in comprehending the information. (Student 23)
**Sociolinguistic aspect**	
Self-positioning	… as a student there, I did not know how actively I could ask for the opportunity to observe a surgery and how I could be involved in the surgery. (Student10)
Relationship with supervisor and other medical staff	My supervisor was very busy with his duties, and I did not know when I could ask him a question. When I did, he spoke too quickly for me to grasp his answer. … I had no idea about how to behave appropriately in this situation in a different cultural context. (Student 13)
Communication with patient	I think that my problem was that I could not conduct medical interviews well by taking the patient’s social and psychological backgrounds into account. (Student 35)
**Sociocultural aspect**	
Different educational culture from their home country	[Student autonomous learning culture] The doctor did not assign tasks to me. Instead, I had to tell him what I wanted to do. As I was unaware of this educational culture, I could not do anything in the first few days. (Student 3)
	[Student responsibility] On the first day, I wanted to observe the doctor during ward rounds, but I was asked abruptly to make a patient note. It was not a SOAP format, and I became nervous because I had no idea about how and what to write on the form. Realising my struggle, a fellow doctor took the patient note from me and said, ‘I can do it.’ I was annoyed by my own uselessness and felt alienated. (Student 17)
	[Different educational systems] The students in North America enrolled in medical school after graduating from college and had already participated in many rotation programs. The responsibilities of local medical students were possibly equivalent to ones of junior residents in Japan … Although we’re all ‘medical students,’ they were much more knowledgeable and had more clinical experience. Consequently, I developed an inferiority complex and lost my self-confidence. (Student 15)
	[‘Competitive’ culture] In the second week onward, some local medical students joined our team … One day, the doctor asked us about the characteristic findings of Crohn’s disease. A student next to me answered immediately while I was still processing the question. It was highly competitive, and I had to invest more efforts into not just improving my English but also my medical knowledge. (Student 36)
Clinical ethics	I was given a chance to be in charge of a patient whose testicular cancer had metastasised to his entire system. Australia had more patients who were informed about their cancer diagnosis compared to Japan. So, at first, I was really worried about how to communicate with a cancer patient in such a situation. (Student 14)

The first aspect of problems/difficulties is the linguistic and communicative aspect related to pronunciation, lexicon, syntax, as well as listening and speaking in conversation. For example, as Student 34 commented, although the participants took a preparatory course at the home institution before the electives, they were overwhelmed by conversation among native speakers in the real setting in terms of the tempo, speed, wording, and expressions. Furthermore, the problem of listening skills led to difficulties in speaking and expressing their opinions. When students were asked a question during the conversation, it was difficult for them to share their opinions and answer the question due to their limited understanding of the discussion so far. Even if they had an opinion, they found it difficult to express the nuances of their thoughts in the second language (Students 6 and 41).

The second aspect of problems/difficulties is concerned with sociolinguistic competence, which relates to knowing who speaks to whom, when, about what, and how (Table 3). Students commented about their self-positioning as international students. Specifically, they had difficulty in understanding the supervisor and institution’s expectations and how they should behave, as international students, in a way that is socially appropriate to the different clinical education cultural context (Student 10). In accordance with that, students were worried about how and when to communicate with their supervisor, particularly in situations where (s)he seemed to be very busy with clinical duties (Student 13). Moreover, students also found it difficult in managing the professional distance to patients and building social relationships with them. For example, Student 35 said that he had no idea about how he could gain a better understanding of the cultural background and psycho-social state of the patients.

The third aspect of problems/difficulties relates to sociocultural competence. Students experienced difficulties caused mainly by different educational cultures and expectations. In the international electives, they perceived that local medical students were given responsibilities and regarded as members of the medical team, and there was an emphasis on autonomous learning, which led to learning environments where students improved themselves through friendly rivalry (Students 3, 17, and 36). Moreover, as Student 14 commented, different stances on clinical ethics caused students’ puzzlement, including the importance of honesty in different cultures.

These linguistic, sociolinguistic, and sociocultural interaction stressors may lead to psychological stressors, such as sickness, loneliness, being homesick, disappointment, and depression. Some students emphasized that the first week was the most difficult time in the international electives due to cultural differences, communication in a foreign language and new learning environments.

### Planning and implementing adjustments in contact situations

Even though students perceived some linguistic and cultural barriers in the international healthcare setting, they wanted to be involved in discussions among health professionals and contribute to patient care. Responding to the identified problems, students planned and implemented adjustments to academic contact situations. This study identified five management strategies they employed, including meaning-focused coping, social relationship building, learning opportunities, language and communication, and approaches to learning (Table 4).

**Table 4.** Planned and implemented adjustments

Theme	Quotations
**Meaning-focused coping**	
Acceptance of cultural diversity	In Canada, many people were from different cultural backgrounds and some spoke English with a strong accent. So, not everyone spoke clearly and in ‘perfect’ English. I saw them communicating with the native speakers confidently. Since then, I realised it was alright to make mistakes while speaking English in such societies. (Student 1)
Language as a tool of communication	I realised that my English didn’t have to be on a par with native speakers. Language was just a ‘tool’ for communication. (Student 7)
**Social relationship building**	
Supervisor and medical staff	I went to an operation room and observed surgical procedures daily. Not only the surgeon but also the other staff members built a rapport with me and willingly supported my learning there. (Student10)
Resident and interns	Initially, I didn’t know how I could be involved in the clerkship. In case of difficulties, a resident provided me with useful advice on my learning there. (Student 5)
Local medical student	By participating in the clerkship with local students, I could learn what the hospital expected students to do and the socially appropriate behaviour in healthcare. I also learned about their lifestyle. (Student 21)
	I joined the track and field club and trained with local students thrice a week. I enjoyed club activities with local students. (Student 30)
International student	I could befriend international students from Hong Kong. On the first day, they came to talk to me and asked me to go out for dinner and shopping on the weekend. I could not only share my experiences and feelings during the clerkship with them, but also discuss our future planning freely. (Student 13)
Patient	The patients always encouraged me with phrases like, ‘You’ll be all right,’ and ‘Enjoy Montreal!’ This motivated me to actively participate in the electives. (Student 1)
**Management of learning opportunities**	
Seizing opportunities	Initially, I didn’t know how I could ask for the opportunity to be involved in the surgery. The second week onward, I started introducing myself to all the medical staff members each time we met, and I tried to tell them what I wanted to do during the surgical operation. The staff did their best to accommodate my requests. (Student 10)
Making alternative plans	In the second week, my supervisor was replaced by someone who was very busy. When he was busy, I sought other learning opportunities, like training in the skill lab or discussing patient problems with residents. (Student 13)
	Every morning, I could choose a supervisor whom I wanted to observe. Hence, on days when I felt tired, I chose a doctor who would finish his clinical education before lunch time. It was important in terms of my time management. I could spare time to read some papers the doctors recommended. (Student 6)
**Language learning and communication management**	
Request for repetition	I tried to ask the native speakers to repeat themselves until I understood them fully. … this tactic helped me get used to communicating in English, and I could talk to people in English without hesitation. (Student 28)
Showing intention of speaking	I think that if you stay silent and do not ask any questions, you might be regarded as a demotivated person. So, in the outpatient ward, I tried to actively share my opinions and ask questions even if I didn’t know how to express in English exactly. (Student 9)
Non-verbal communication	I tried to maintain eye contact with people while conversing in the second week. I realised that facial expressions conveyed a lot of information, and eye contact was essential while communicating. (Student 29)
Creating a relaxed, safe environment in patient encounter	When I conducted a medical interview with a child and his mother, I was mindful about maintaining a smile. Creating a friendly atmosphere is important. I began with general conversations, such as the kid’s school life, before taking the history. (Student 15)
Self-study strategies	Communicating with local people, I tried to memorise the useful expressions they used. Then, I looked them up and used them in the following conversations. (Student 5)
	Whenever I talked to myself, I tried to speak in English. This was a good way to practice for my English. I took note of useful phrases from conversations between native speakers and repeated them aloud to myself. (Student 38)
**Developing approaches to learning**	
Use of different channel	If you are quiet, no one helps you. So, at least, I tried to ask questions until I understood the diagnostic name. Even when I couldn’t understand what they discussed, I requested them to write the name down on my note and studied this disease. (Student 41)
Emphasis on learning through interaction	I tried my best to ask the doctor questions. He tried to understand what I wanted to know and answered my questions kindly. Since then, I could ask whatever I needed to during ward rounds. I enjoyed communicating with the doctors. (Student 28)
Speech shadowing	I repeatedly shadowed the recorded voice of doctors during CT interpretations to practice English. This was effective because I was gradually getting better at commenting in English in CT interpretations. (Student 35)
Development of learning cycle	I went to the patients’ bedside and took their medical history and conducted physical examination in English. I made my patient notes based on information that I elicited. Subsequently, I compared these with the doctor’s patient notes to identify the areas where I needed to improve. I did this regularly. (Student 7)

Meaning-focused coping was fundamental to their subsequent participation in the international electives. It involved active cognitive restructuring related to cultural diversity and the nature of cross-cultural communication. Specifically, through observing communication styles among people from different cultural backgrounds, students noticed that sharing opinions and mutual understanding are pivotal to multicultural society; this observation helped them realize they could communicate with people without being afraid of making mistakes (Student 1). Additionally, their perceptions of language changed. For instance, students regarded language as a tool of communication, and started thinking that they did not have to speak perfectly like native speakers (Student 7). Acceptance of cultural diversity and changes in their perceptions of language made them feel more comfortable and led to more active engagement in the electives.

Establishing better relationships with people is essential to gaining social support. Students tried to broaden their social network with supervisors, medical staff, residents, interns, local medical students, international students, host families, and patients. For example, Student 10 had an opportunity to learn surgical procedures from clinical staff members. Student 21 learned about how medical students should position themselves in hospitals from local medical students.

Managing learning opportunities includes seizing opportunities and constructing alternative plans. Students tried to gain learning opportunities about topics such as surgical procedures, history taking, and case presentation, because they realized that gaining meaningful learning from their international experiences would increase their active engagement (Student 10). They emphasized the importance of not hesitating to tell the supervisor and medical staff what they wanted to do during the electives. As such, relationship building in hospitals has a great deal to do with management of learning opportunities. Students 6 and 13 also employed avoidance as a learning strategy. For example, Student 13 made an alternative plan to learn in the skills lab instead of asking for the doctor’s teaching when (s)he was busy.

Language learning and communication management was the response to the linguistic and communicative aspect of problems/difficulties. Students 9, 15, 28, and 29 commented that they tried to find a way to be involved in communicative events, such as discussions, case presentations, and daily conversations with people in the local context. For example, in order to fully understand what people were saying, Student 28 stopped hesitating to ask for repetition, confirmation, or clarification in conversation. Student 29 realized that non-verbal communication strategies, including facial expressions, eye contact and gestures, were useful for effectively conveying what they wanted to say. Moreover, as Students 5 and 38 commented, they reconsidered their approaches to language learning and adopted new techniques, such as noting useful phrases and expressions in actual conversation between local people.

For academic management, as Students 28, 35 and 41 commented, they adopted new approaches to clinical learning emphasizing the importance of learning through social interactions. For example, when Student 28 directly asked the doctor a question, he responded kindly and in a comprehensive way. This experience changed the student’s perception of learning. Up to that time, due to language difficulties, the student had focused on self-study through reading. However, following his conversation with the clinical teacher, the student believed that learning through discussion was more effective. Student 41 asked doctors to write the medical terms down in his notes when he missed what they had said. In other words, the student used, if necessary, a different communicative channel (i.e. not oral but written) to fully understand their conversation.

## Discussion

The present study aimed at exploring medical students’ learning experiences during international electives. The findings demonstrate that students perceived various learning outcomes from cross-cultural experiences even in the short-term programme, such as medical knowledge and skills, communication, and personal development. There are some variations in the descriptions of students’ experiences and perceptions in their e-portfolios depending on the country visited and specialty rotated; however, there were similarities in their learning trajectories, including perceived outcomes, difficulties encountered and adjustment strategies. These findings are congruent with a previous study exploring undergraduates’ perceptions of learning outcomes in short-term international courses in liberal arts[5]. The present study has added to the existing knowledge in that it fully describes the processes by which students perceived learning experiences positively during a short-term international elective course. Specifically, this study revealed that even though this elective course lasted only four weeks, the processes of student learning through cross-cultural experiences involving language and cultural management in contact situations included the full range of responses: from the stage in which deviations from norms occur to the stage in which adjustments are implemented[24].

Previous experiences of clinical clerkship in students’ home country are pivotal to gaining more meaningful learning in the international electives. In entering a foreign cultural context, people are able to see themselves more objectively by comparing their previous learning experiences in their home country with the new cross-cultural experience[27]. For instance, the students in this study made new meaning in relation to international healthcare, career management and development, and medical education as learning outcomes of the international electives through comparison between home and foreign cultures. Based on their previous experiences in their home country, new meaning was produced through a process of ‘making the familiar unfamiliar’[28].

Language management theory in academic contact situations [24] is a powerful analytical lens for exploring the students’ learning experiences in the international electives. The ways that the students participated in the international electives were socially and culturally dynamic processes and frequently changed as a result of their planning and implementation of adjustments towards perceived problems. This contradicts the W-curve or U-curve model of transition, which shows a predictable pattern of sojourners and international students’ cultural adaptation [29,30]. The stages outlined in this model are useful for understanding a general picture of their learning trajectory in the cross-cultural setting. However, they do not allow the researcher to fully describe the complex phenomena of their learning experiences in cross-cultural settings from a fine-grained analytical perspective.

Managing problems in academic contact situations is not a linear process; it is iterative and cyclical[31]. For example, at first, a student asked local health professionals to write down clinical terms which she could not understand during a discussion about a patient’s problem, and she studied medical knowledge related to these terms independently after the clinical education session. However, she noticed the limitations of self-study and started to ask her supervisor questions directly. That is to say, the student changed her adjustment strategies to using different interaction channels, from self-study to direct communication for interactive learning in situ. Moreover, it should be noted that one adjustment strategy did not always correspond to one particular problem/difficulty. In other words, students employed several strategies, such as meaning-focused coping, social relationship building, and management of learning opportunities, to overcome a particular problem. Therefore, the management process is a context-dependent and complex phenomenon.

Communication in a foreign language is one of the most challenging issues for the international elective students, where academic activities mainly consisted of language-mediated social practices. Although all students met the minimal foreign language requirement of the host institution, many students perceived themselves as quiet and relatively passive learners in the first week. Almost all noted language-related issues during their clinical clerkship in an overseas hospital. In fact, behind their reticence were multiple, interrelated issues including grammatical, sociolinguistic, and sociocultural aspects of communication. The ‘language barrier’ is attributed not only to linguistic limitations but also issues of culture, identity, pedagogy, and power[32]. In particular, for international students from a ‘monolingual’ community such as Asian countries, the major issue is related to not linguistic knowledge but language use in different cultural contexts, due to their relatively limited opportunities for social interactions with people who speak other languages.

Given this, building social relationships in the new cultural context is fundamental to overcoming the problems deriving from interactional stressors. Development of social networks allowed students to seize learning opportunities, understand expectations from the host institution, and gain social and emotional support from others. For example, Student 30 could socialize with local students by exploring his personal interests and participating in activities in the local community (i.e. at a track and field club). However, at the beginning, many students placed themselves in inferior social positions mainly due to their perceived foreign language limitations, which hindered them from actively learning medicine in a clinical setting overseas[33]. Additionally, the lack of social affiliation in the host community promoted their sense of isolation[23]. In this situation, meaning–focused coping was essential because it functioned as a cognitive reconstruction of the social world and self[19]. For instance, students started to perceive language as a means of communication and to accept cultural diversity in the social world, and could therefore place themselves in an equal position to others in the new cultural context. These perceptual changes drove their social relationship building to manage participation and obtain membership in a social group.

The students in this study tried to build a support network and shared their problems and feelings with people, including other Asian international students, host families, and young physicians. This adjustment strategy functioned as effective emotional control. Contrary to these findings, previous studies in higher education reported that Asian international students tend to think that they should be able to handle emotional distress independently and their favoured coping style is emotional suppression [14,34–37]. Asian cultural norms such as shame and loss of face (i.e. embarrassment) resulted in reluctance to seek help because it implies personal failure to manage emotions[37]. The findings of the present study about Japanese students’ emotional coping, which differ from previous studies, might be caused by the learning context in terms of their length of stay, the size of community where they participated, and expectations of the host institution. In other words, their participation in cross-cultural activities was socially situated. In this study, students participated in the short-term programme in a clinical setting which was a relatively small community and required them to communicate with others, such as healthcare team members, local peers, patients and so forth. Building social relationships was essential for them to obtain better learning experiences in the four-week programme. Therefore, the learning context is key to exploring international students’ acculturation processes.

Our findings suggest that active cognitive reconstruction of the perceived meaning of the situation (i.e. meaning-focused coping) is an important strategy for achieving meaningful learning in an international experience[38]. It is true that their language-related competency could have been further improved at the pre-departure stage in the home institution. However, more importantly, the educators at the home institution need to offer more opportunities for students to consider the nature of cross-cultural communication in multilingual and multicultural societies, discuss useful learning and coping strategies to respond to problems they encounter, and clarify the goals of participating in international electives. For example, for the home institution, in the preparatory education, graduates who had experienced international electives would be invited as lecturers to facilitate discussions on potential cultural issues and survival tips during their overseas stay. Offering more online learning opportunities with students and educators from partner host institutions at the pre-departure stage could be useful for the students to understand the context of healthcare in their target location and promote relationship building in advance. One relevant educational strategy is arranging homestay opportunities for international students, which can influence language learning, cultural immersion, and development of professional skills for health science careers[39]. For clinical supervisors at host institutions, it is important to create an environment which does not increase international students’ sense of isolation and to help them broaden their social networks in the host community, including supervisor-student relationships, which would become sources of social support for international students [13,36,40].

Responding to the unforeseen current situation of the COVID-19 pandemic, we had to develop our practices to fit within the boundaries of what was, and was not, allowed or possible (e.g. with limited clinician availability)[41]. Educational opportunities for online cross-cultural interaction will increase, while, unfortunately, face-to-face opportunities may decrease. From the perspective of cross-cultural adaptation in international programmes, both synchronous and asynchronous online interactions across cultures in medical education will be a new research agenda[42].

In terms of our study’s limitations, it should be noted that most of the research team had experience studying abroad and the results, particularly at the coding and data interpretation stage, could have been affected by experimenter bias. Moreover, regarding the validity and reliability of the data analysis, this study did not conduct member checking to ensure that the interpretation of their experience was accurate. As the results were based on qualitative analysis of a relatively small number of participants from one educational institution, selection bias might also have affected the data collected. This is because the students are likely more willing to acquire global competence and report more positive experiences in cross-cultural contexts.

Although self-evaluation could yield important data, further study is needed using quantitative measure tools, such as a rating of their performance by the host institutions, to indicate statistical significance regarding their learning in the electives. From a longitudinal perspective, it would also be worthwhile to examine how their international experiences in undergraduate education influence their professional identity formation and actual career development. Exploring the perspectives of not only students but also clinical supervisors working with international students at host institutions is another important issue to be addressed. By investigating the supervisors’ perspectives, the pedagogical implications of the supervisory role can be revealed, including provisions of support, teaching strategies, difficulties and coping behaviour of teachers.

## Conclusion

In line with the saying of Steve Jobs, ‘the journey is the reward’[43], this study clarified that students’ perceived learning outcomes of their international electives cover not only language/communicative aspects but also clinical, cultural, and social aspects. Moreover, with a focus on the process of their learning experiences, we revealed what problems they encountered and how they overcame them during the electives. Therefore, this study provides new insights into student participation in short-term international programmes to maximize their clinical learning and to develop academic and social support strategies for educators at both home and host institutions.
